# On the Nature of Evidence and ‘Proving’ Causality: Smoking and Lung Cancer vs. Sun Exposure, Vitamin D and Multiple Sclerosis

**DOI:** 10.3390/ijerph15081726

**Published:** 2018-08-12

**Authors:** Robyn M. Lucas, Rachael M. Rodney Harris

**Affiliations:** 1 National Centre for Epidemiology and Population Health, Research School of Population Health, The Australian National University, Canberra 2600, Australia; rachael.rodney@anu.edu.au; 2 Centre for Ophthalmology and Visual Science, University of Western Australia, Perth 6009, Australia

**Keywords:** epidemiology, causality, association, smoking, lung cancer, vitamin D, sun exposure, multiple sclerosis

## Abstract

If environmental exposures are shown to cause an adverse health outcome, reducing exposure should reduce the disease risk. Links between exposures and outcomes are typically based on ‘associations’ derived from observational studies, and causality may not be clear. Randomized controlled trials to ‘prove’ causality are often not feasible or ethical. Here the history of evidence that tobacco smoking causes lung cancer—from observational studies—is compared to that of low sun exposure and/or low vitamin D status as causal risk factors for the autoimmune disease, multiple sclerosis (MS). Evidence derives from *in vitro* and animal studies, as well as ecological, case-control and cohort studies, in order of increasing strength. For smoking and lung cancer, the associations are strong, consistent, and biologically plausible—the evidence is coherent or ‘in harmony’. For low sun exposure/vitamin D as risk factors for MS, the evidence is weaker, with smaller effect sizes, but coherent across a range of sources of evidence, and biologically plausible. The association is less direct—smoking is directly toxic and carcinogenic to the lung, but sun exposure/vitamin D modulate the immune system, which in turn may reduce the risk of immune attack on self-proteins in the central nervous system. Opinion about whether there is sufficient evidence to conclude that low sun exposure/vitamin D increase the risk of multiple sclerosis, is divided. General public health advice to receive sufficient sun exposure to avoid vitamin D deficiency (<50 nmol/L) should also ensure any benefits for multiple sclerosis, but must be tempered against the risk of skin cancers.

## 1. Introduction

**Figure 1:**
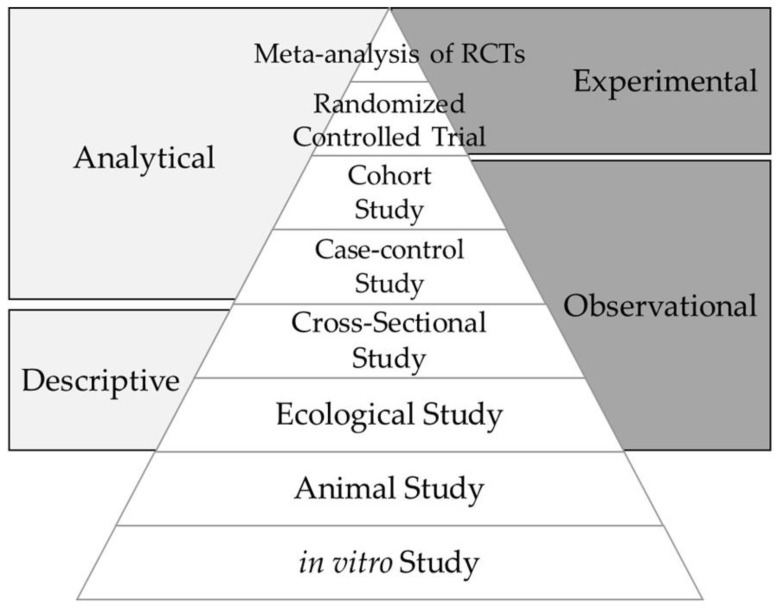
The hierarchy of evidence, increasing from the base of the pyramid to the gold standard for establishing causality.

Epidemiological studies seek to determine the association between an exposure and a health outcome. The strongest evidence, particularly required for therapeutic studies, derives from a systematic review (with meta-analysis) of well-conducted randomized controlled trials (RCTs) [1]. However, such trials are not always feasible to assess whether environmental exposures causally alter disease risk. For studies of etiology, a systematic review of prospective cohort studies is considered the highest level of evidence [1], but again, such studies may not be feasible, for example, for rare diseases. Instead, the assessment of causation may need to be inferred from the ‘harmony of evidence’—the accumulation of evidence from different, including multidisciplinary, approaches that consistently tells the same story. Figure 1 shows a commonly used schematic of the hierarchy of study types contributing evidence for human health.

Beginning at the base of Figure 1, in vitro studies typically focus on specific cells (e.g., immune cells) that are ‘exposed’, often by bathing them in a chemical solution. This is a highly artificial situation, but can provide supporting mechanistic evidence, although care needs to be taken that the ‘exposure’ is at a realistic physiological, rather than therapeutic, concentration. For example, early in vitro studies of vitamin D and immune cell function treated immune cells with calcitriol (the active form of vitamin D) at concentrations around 1000 times higher (nanomolar) than that seen in human blood (in the picomolar range) [2].

Animal models allow control of both genetic variation and environmental exposures, and invasive studies can be undertaken that are not possible in humans. In addition, shorter lifespans allow the effects of early life exposures on adult disease to be tested within a relatively short period of time. Such studies have been invaluable in understanding biological pathways, for example from environmental exposure, through epigenetic change, to disease risk [3]. However, findings from animal models commonly do not translate into human studies [4]; as such animal studies provide supporting evidence, but are not sufficient to infer causality.

Epidemiological evidence commonly proceeds from observation of geographic and/or temporal patterns of disease incidence at the level of populations (ecological studies) that generate hypotheses, to individual-level studies testing whether the putative risk exposure is associated with an increase (or decrease) in disease risk. Human studies are inevitably ‘noisy’—individuals are not genetically homogeneous, and few exposures exist in isolation (for example, the physically inactive may be more likely to smoke, eat a poor diet, be overweight, and consume more alcohol), making it difficult to delineate the specific exposure that alters disease risk. In addition, there are challenges with falling participation rates in research studies, leading to a risk of selection bias (are those who participate in a study different in some important way to those who refuse to participate?), long time frames, and high costs.

Here we use the case of smoking, as a risk exposure that is well-established as the major cause of lung cancer, to compare and test the evidence suggesting that low sun exposure and/or low vitamin D status ‘causally’ increase the risk of the autoimmune neurological disease, multiple sclerosis. We begin by briefly describing the two outcomes—lung cancer and multiple sclerosis—and the two exposures of interest—cigarette smoking and low sun exposure/vitamin D.

## 2. The Outcomes: Lung Cancer and Multiple Sclerosis

Lung cancer was a rare disease in the early 20th century; for example, in a publication from 1912, “*On one point, however, there is nearly complete consensus of opinion, and that is that primary malignant neoplasms of the lungs are among the rarest forms of disease*” [5]. Lung cancer is now the leading cause of cancer death in many developed countries, and, in Australia, is the fifth most common cancer diagnosed [6]. The most common type is non-small cell lung cancer, including adenocarcinoma, squamous cell (epidermoid) carcinoma, and large cell undifferentiated carcinoma [6]. Lung cancer is typically a disease of older age, with a mean age of onset of around 70 years [7].

Multiple sclerosis (MS) is an inflammatory and neurodegenerative disease of the central nervous system. It is the commonest disabling neurological disease of young adulthood after trauma [8], with an age of onset most commonly between 20 and 50 years [9]. MS is widely considered to be primarily an autoimmune disease, where there is an immune attack on ‘self-antigens’ in the myelin sheath of nerve axons [9]. The resulting slowing of nerve conduction, and ultimately degeneration of axons, leads to (most commonly) repeated bouts of neurological dysfunction, followed by progressive disability.

## 3. The Exposures: Tobacco Smoking and Low Sun Exposure/Vitamin D

Smoking of tobacco in cigarettes became increasingly common from the late 19th century when the manufacture of cigarettes became automated and costs fell. Tobacco smoke contains more than 5000 chemicals, many of which are known to be toxic and carcinogenic [10]; these are delivered directly to the lung through smoking.

Solar radiation includes infrared, visible and ultraviolet (UV) wavelengths. Ultraviolet radiation is further broken down into UV-C, UV-B and UV-A, according to wavelength; the shortest wavelengths (UV-C and most of the UV-B) are filtered out in the stratosphere. Thus UV radiation at Earth’s surface is predominantly UV-A (>95%) with the remainder UV-B. The UV waveband has been considered the most important for human health, with over-exposure the main cause of skin cancers and chronic eye diseases such as cataract (reviewed in [11]). Exposure of the skin to UV radiation also causes both local and systemic immune suppression (reviewed in [12]). UV-induced immune suppression is important for the development of skin cancer, and also allows the reactivation of latent viral infections. By stimulating a more tolerant immune milieu, UV-induced immune suppression may reduce the risk of autoimmunity [13]. Exposure of the skin to UV radiation in the UV-B (280–315 nm) wavelengths is the major source of vitamin D in many populations, and vitamin D has known immunomodulatory effects, that could plausibly reduce the risk of MS [14]. Vitamin D status is assessed according to the concentration in blood of a metabolite, 25-hydroxyvitamin D (25(OH)D). The major determinant of the 25(OH)D concentration in many locations is recent sun exposure [15,16], so that 25(OH)D concentration is a measure of both vitamin D status and recent sun exposure.

## 4. Ecological Studies Provide the First Links between Exposures and Outcomes

**Figure 2:**
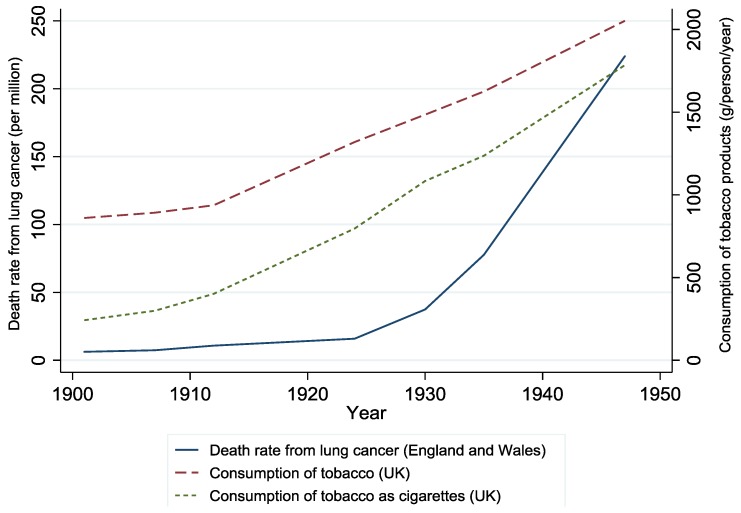
Death rate from lung cancer and consumption of tobacco and cigarettes over time in the United Kingdom (redrawn using data from [17]).

The first indication that smoking may increase the risk of lung cancer came from ecological studies, showing that mortality rates for lung cancer in the UK began to increase from approximately 1925, around 15 years after the increase in consumption of tobacco and cigarettes (Figure 2) [17]. A similar pattern has been repeated over time and location—rising prevalence of smoking is mirrored by rising prevalence (and mortality) of lung cancer [18].

**Figure 3:**
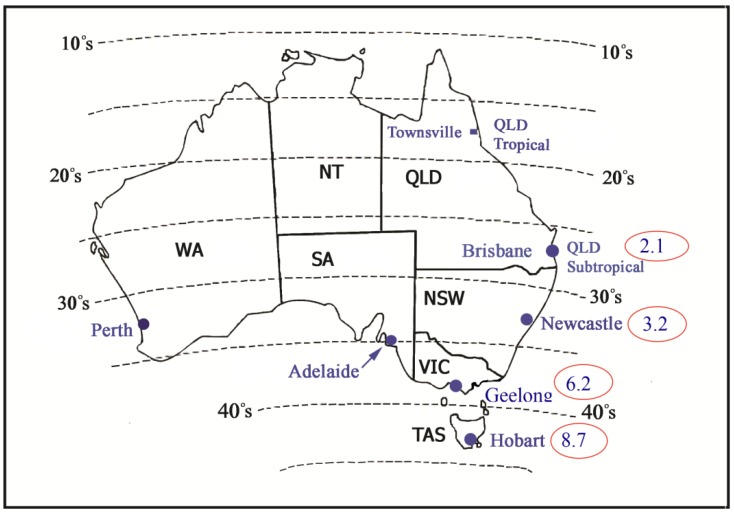
Incidence of a first clinical diagnosis of central nervous system demyelination, as a marker of multiple sclerosis incidence, in Australia in 2003–2006, based on data from the Ausimmune Study [20] (dotted lines represent latitude, annual incidence (per 100,000 population) is circled).

A link between low sun exposure/vitamin D and increased risk of MS was first hypothesized, not on recognition of similar temporal patterns, but on a striking geographical pattern. One of the best-known and unusual characteristics of MS epidemiology is that the incidence and/or prevalence increases with increasing distance from the Equator in both northern and southern hemispheres [19], including within relatively racially homogeneous populations [20], see Figure 3. Such a latitude gradient suggests a risk exposure that also varies with latitude; one that has captured attention and led to further study is variation in ambient UV radiation [21], particularly through the link between UV radiation and vitamin D [22].

**Figure 4:**
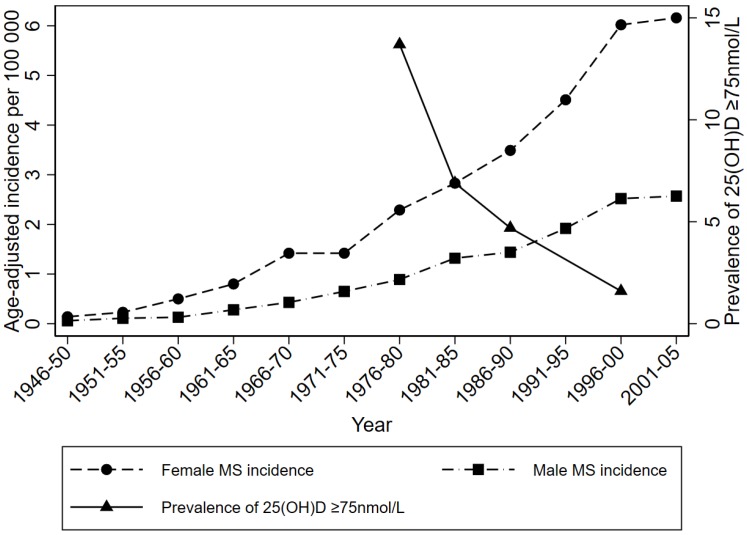
Data from Sweden on age-adjusted incidence (per 100,000 person years) of MS overlaid with the prevalence of 25(OH)D level ≥75nmol/L (plotted from data available in [24] and [25]) in time periods from 1946–2005.

However, temporal changes in incidence also provide some support to an association between low sun exposure/vitamin D and higher risk of MS. The incidence of MS is thought to have been increasing over the last 50 years, although changes in diagnostic criteria account for at least some of the reported increase. Nevertheless, in well-defined locations where efforts have been made to replicate case ascertainment across repeated studies, incidence of MS does seem to have increased [23]. It has been difficult to objectively track 25(OH)D concentrations over time, because of high variability in laboratory assays. However, recent studies from Sweden show rising MS incidence [24] over a similar time course as decreasing 25(OH)D levels [25] in blood samples that had been stored and analyzed in a single laboratory at one time point (Figure 4).

Ecological studies—correlations between population-level patterns of exposure and disease—can provide the first indications of links between exposures and disease [26], and may be the only feasible study type in some cases, e.g., understanding health risks of global warming. However, they are relatively low on the hierarchy of evidence (Figure 1). This is due to the ecological fallacy: “*an association observed between variables on an aggregate level does not necessarily represent the association that exists at an individual level*” [27]. A good example of this is the marked correlation between per capita chocolate consumption and the number of Nobel Laureates per 10 million population [28], but there are many others, where the correlation is clearly highly unlikely to reflect causation.

## 5. Individual-Level Studies to Better Define Associations

Individual-level observational studies ask either, did those with the disease have less/more exposure than those without the disease (case-control study), or did those with more/less exposure get the disease more often than those without/with less exposure (cohort study). Importantly, to be a cause of increased disease risk, the exposure must always precede the outcome. This is clearer in cohort studies, where the exposure is typically measured first, and the participants followed over time until disease occurs. This exposure–disease relationship can also be clear in case-control studies where newly diagnosed (incident) cases are recruited, but may be less clear when cases have had the disease/health outcome for some time (prevalent cases). Establishing the correct temporal relationship between exposure and outcome is particularly a problem in cross-sectional studies, or surveys; indeed, this study design is descriptive rather than analytical and poorly suited to assess whether an exposure causes a health outcome.

Case-control studies can be completed relatively quickly because the cases have already accrued. These studies are typically less expensive than cohort studies and may generate the evidence required to support the need for a cohort study. In their 1947 case-control study, Doll and Hill recruited hospital patients diagnosed with lung cancer (cases), and patients at the same hospital, matched on sex and age within 5 years with a non-lung cancer diagnosis (controls); they detailed the smoking history of participants in both groups [17]. Most men in both groups smoked, but the prevalence was higher in cases than controls (99.7% cases; 95.8% of controls; *p* = 0.00000064) and cases smoked more cigarettes for a longer duration (i.e., higher dose of exposure) than controls. Their conclusion from the study was that *“there is a real association between carcinoma of the lung and smoking”* [17]. They went on to study the association between smoking and lung cancer in the 1958 British doctors cohort study [29], where they concluded: “*we have found death rates per 1000 per annum from cancer of the lung of 0.07 in non-smokers, 0.93 in cigarette smokers, and 2.23 in cigarette smokers of 25 or more cigarettes a day….we can say that the death rate of cigarette smokers from cancer of the lung has been thirteen times the rate of non-smokers, and that the death rate of heavy cigarette smokers has been over thirty times the rate of non-smokers*….”

The interrogation of sun exposure/vitamin D as risk factors for MS similarly proceeded to individual-level studies. Several case-control studies showed a protective association between higher sun exposure and lower risk of MS [30,31] or a precursor state (first demyelinating event; FDE) [32,33]. The evidence is consistent across a range of markers of higher sun exposure: winter residential UV radiation [34], self-reported past time outdoors, with or without adjustment for ambient UV radiation levels [30,31,32,33], cumulative actinic damage to the back of the hand measured using silicone skin casts [31,32], cumulative UV-related disease score (skin cancers, cataracts, pterygium) [32], and 25(OH)D levels close to the time of diagnosis [32,33]. The reduction in risk is significant, for example, 55% lower risk with high (score > 3) compared to low (≤3) cumulative actinic damage [32].

MS is uncommon—global incidence of 0.93 per 100,000 [35] compared to that of lung cancer of 28 per 100,000 [35] (and this is much lower than the mortality rate noted by Doll and Hill above of 93 per 100,000). Case-control studies are particularly suited to the study of uncommon diseases such as MS, as the study begins after identifying cases. For uncommon diseases, it is unlikely that there will be sufficient new cases occurring within a reasonable length of time to be able to use a cohort study design, even with a large sample size. For such diseases, a hybrid model is used—a case-control study nested within a cohort study. Exposures are measured on the full cohort at study entry (and possibly multiple time points), and cases develop the disease during the period of follow-up. Controls are drawn from the cohort members who have not developed the disease, and the analysis is as for a case-control study. In a very large US cohort, the risk of MS was significantly lower in participants in the highest quintile of 25(OH)D levels compared to the lowest—a 60% lower risk in those with 25(OH)D levels ≥99.2 nmol/L compared to <63.2 nmol/L [36]. There was not a clear dose-response, but rather a significant difference only between the highest and lowest quintiles.

## 6. When in Life Does the Relevant Exposure Incur Risk?

For smoking, early age of starting to smoke is associated with increased risk of developing lung cancer [37], but this seems to be primarily a dose effect, with those starting younger also smoking more heavily and for a longer overall duration. Lung cancer occurs following an accumulation of damage caused by the toxins and carcinogens in tobacco smoke.

For MS, it is not clear when the disease process starts, and thus when the relevant exposure is important [38]. There is evidence for protective effects of higher ambient UV radiation *in utero* [39,40], in childhood [30,31], and in adulthood, including close to diagnosis [32,36]. There are plausible explanations for these effects, with the timing of the protective *in utero* exposure coinciding with major periods of immune system development [39], childhood exposures potentially modulating the specificity of adaptive (memory) immune responses to ‘childhood’ viral infections, and adult exposures suppressing adaptive immune responses, to ‘hold off’ the over-reaction that results in immune destruction of myelin and the onset of MS [41]. This question of the relevant timing of the exposure complicates the consideration of causality.

## 7. Sun Exposure or Vitamin D?

Observational studies have used past sun exposure as a marker of past vitamin D status, while 25(OH)D levels, e.g., those measured in cohort studies [36], derive primarily from higher sun exposure. Animal studies suggest both exposure to UV radiation (without increase in 25(OH)D) [41] and treatment with vitamin D metabolites [42] reduce the risk of experimental allergic encephalomyelitis (EAE), the animal model of MS. In human studies, the effects of sun exposure and 25(OH)D were found to be statistically independent, showing benefits of both higher sun exposure and higher 25(OH)D levels [32]. Indeed, sun exposure induces immune modulatory effects through both vitamin D-dependent, and vitamin D-independent, pathways [13,43].

In a recent multi-ethnic case-control study, higher sun exposure was associated with reduced risk of MS (or FDE) across Whites, Hispanics, and Blacks, while higher 25(OH)D levels were associated significantly with reduced risk of MS/FDE only in the White population [33]. This lack of consistency for 25(OH)D levels across races seems biologically implausible, but may be explained by the weaker correlation between recent sun exposure and 25(OH)D levels in darker skinned, compared to lighter skinned, individuals [33], i.e., 25(OH)D reflects recent sun exposure, rather than vitamin D per se. Nevertheless, a nested case-control study within a large cohort study showed that higher total vitamin D intake (from diet and supplements, when supplements were assumed to contain 400 IU of vitamin D) was associated with reduced risk of MS [44]. Dietary intake alone was not significant and it is possible that some other factor associated with taking supplements may have confounded the results [44]. Mendelian randomization studies show that genetically higher 25(OH)D levels are associated with a reduced risk of MS [45,46], although the genetic variants used in these studies account for only around 5–9% of the variance in 25(OH)D levels [47], or <5 nmol/L [48].

## 8. From Observational Studies to Randomized Controlled Trials

The importance of unpicking vitamin D vs non-vitamin D benefits of sun exposure is that the former may be able to be replicated using vitamin D supplementation. To date, vitamin D supplementation studies in people with MS have shown limited benefit (reviewed in [49,50]), confined to fewer new gadolinium-enhancing (inflammatory) lesions seen on magnetic resonance imaging and alterations in immune parameters, but with little evidence of improvement in clinical disease parameters. Nevertheless, in MS epidemiology, risk factors for onset appear to be different to those of progression and disease activity [51]. The strongest evidence for a role of vitamin D is in relation to MS risk, but vitamin D supplementation studies have been in people with MS with the outcome of interest, disease progression. New studies are underway to test vitamin D supplementation for the prevention of MS onset following a FDE (e.g., the PrevANZ Study in Australia (Australian New Zealand Clinical Trials Registry ACTRN12612001160820), and the D-Lay Study in France (Clinicaltrials.gov, NCT01817166)), as being more closely related to ‘risk’. In addition, results of a small clinical trial of UV-B phototherapy have recently been released [52]. At 12 months, there was a 30% reduction in conversion to MS following a FDE in those who received an 8-week course of UV-B phototherapy compared to those who did not; however the sample size was very small (n = 10 in each arm) and the result was not statistically significant.

The trials currently underway or recently completed aim to prevent onset of MS after a FDE—but this may not be the same as preventing the onset of the FDE, i.e., it may not be possible to stop the disease after this first manifestation of immune-mediated demyelination. True prevention trials in MS are not feasible—it is not clear what age at intervention is most appropriate or effective, e.g., *in utero*, in childhood, in adulthood; and MS is an uncommon disease that manifests most commonly in young adulthood (30–40 years), so that a very large sample size followed for a very long time (who have high compliance with the intervention) would be needed.

We have become focused on RCTs as the highest level of evidence as they should minimize bias and confounding, and the intervention clearly precedes the health outcome; they should deliver a conclusion on causation. There are, however, a range of issues with RCTs, including that they typically involve non-representative study samples and have a (relatively) short duration [53]. Indeed, the major health gains in the last 50 years have been based (largely) not on RCTs, but on observational evidence. Cardiovascular disease (CVD, including ischaemic heart disease and stroke) is the major cause of death globally, accounting for a combined 15.2 million deaths in 2016 [54]. The risk factors for CVD—smoking, high blood pressure, high cholesterol—were deduced from observational studies. Primary prevention for these risk factors—based on observational evidence—has led to major reductions in CVD risk and mortality [55].

## 9. From Association to Causation Using Evidence from Observational Studies

Observational studies must be undertaken rigorously, with a study design that is the highest in the evidence hierarchy that is feasible for the disease and exposure under consideration, ensuring an adequate sample size to minimize the risk of chance, careful sample selection and measurement of exposures and outcomes to minimize the risk of bias and ensure that the results are generalizable to the population of interest, and careful consideration and measurement of potential confounding factors. The entirety of the evidence should be in harmony—Bradford-Hill formalized this idea through his ‘criteria for causality’ which he described as an ‘aid to thinking’, initially to approach smoking as a risk factor for lung cancer [56] (see Box 1).

Box 1 **Bradford-Hill’s aids to thinking about causality, with comment on sun exposure/vitamin D and risk of MS.**

Temporality: the exposure must precede the health outcome. There is convincing evidence that low sun exposure/vitamin D precede the development of clinical signs of MS.

Strength of the association: a strong association is more likely to be causal than a weak one, as it is less likely to be attributable solely to uncontrolled residual confounding. The associations between sun exposure/vitamin D and MS are relatively weak, compared to that of smoking and lung cancer.

Consistency: a causal explanation is more likely if a similar result is found in different study designs and in different populations. There is consistent evidence across a range of study types and populations that low sun exposure/vitamin D are risk factors for MS.

Specificity: if an association is limited to or greatly increased in specific groups with a particular environmental exposure, it is more likely to be causal, e.g. does wearing of a bicycle helmet reduce all types of injury, or specifically head injury? Mendelian randomisation studies show a significant reduction in MS risk in association with genetically higher 25(OH)D levels.

Biologic gradient/Dose response: evidence of increasing risk with increasing exposure supports a causal relationship, but is not required, for example, there may be a threshold effect. This is apparent for sun exposure but not for 25(OH)D where a threshold relationship may exist.

Plausibility: while biological plausibility strengthens the causal argument, it is dependent on the knowledge of the day, and plausibility may be argued based not on evidence but on beliefs. There are plausible pathways from higher sun exposure/25(OH)D to reduced risk of MS through modulation of immune function.

Experiment: evidence from animal studies or intervention trials can support a causal association; but animal models may not fully replicate human disease and not all associations are amenable to testing in intervention trials. Animal studies support a causal effect of high sun exposure/vitamin D and reduced risk of MS. Vitamin D supplementation studies show little clinical benefit in people with MS.

Analogy: as for plausibility, this can be argued based less on evidence than on beliefs, but can be used to support an otherwise weak association. For example, similar findings of low sun exposure/25(OH)D levels as risk factors for related autoimmune diseases with similar immunopathology, such as type 1 diabetes, strengthens the argument for MS.

Coherence: does it all fit together? Are the results in harmony?

We now have additional tools to contribute evidence particularly around plausibility, and to better understand consistency, or lack of consistency, for example according to genotype. Risk may be stratified according to genotype, and differential effects by genotype may provide evidence to clarify a causal pathway, e.g., genetic variation in the α5 nicotine cholinergic receptor subunit (CHRNA5) gene increases the risk of smoking-related lung cancer, and predicts smoking cessation [57].

Epigenetic changes caused by environmental exposures, and seen in disease states, can serve to better link the exposure to the outcome, enhancing causal inference [58]. For example, cigarette smoking has widespread effects on the epigenome of the developing fetus, and may be the conduit from maternal smoking to intrauterine growth retardation [59], as well as effects in later life on memory and mood (depression) [60].

## 10. Are We There yet for Sun Exposure, Vitamin D, and MS?

**Figure 5:**
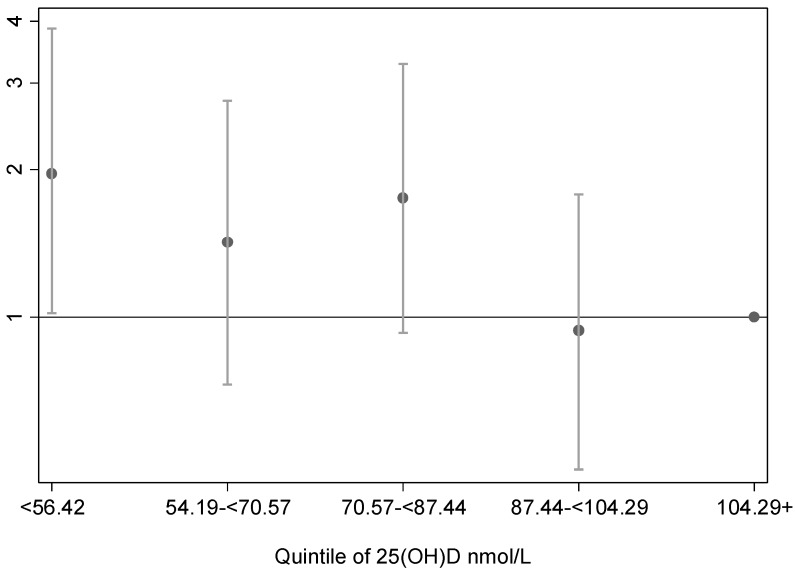
Odds of being a case with a first demyelinating event (a precursor of MS) by quintile of serum 25(OH)D, with the highest quintile as the reference category. Only the lowest quintile of 25(OH)D concentration is associated with a significant increase in risk (replotted from data from the Ausimmune Study [32]).

Animal, in vitro, ecological, and observational studies are relatively consistent in supporting an association between low sun exposure/25(OH)D at various life stages, and increased risk of MS. The effect sizes, compared to well-established causal associations such as that for smoking and lung cancer, are relatively small. A dose-response effect is more convincing for past sun exposure than for 25(OH)D, which seems more like a threshold of effect, whereby low levels (<50 nmol/L) are required before there is an increased risk (see Figure 5). The pathway from low sun exposure/25(OH)D to reduction in MS risk is plausible, through modulation of the immune system and a more regulatory immune milieu. In all, the evidence is coherent and in harmony.

Opinions are varied on whether causality is established for higher 25(OH)D levels [61], and/or higher levels of sun exposure [51] as protective of MS. More evidence from observational studies is unlikely to increase the confidence in either low 25(OH)D or low sun exposure being causal risk factors for MS, although RCTs currently underway may provide information about whether supplementation with either vitamin D or UV-B radiation can reduce the risk of progressing to a diagnosis of MS following a FDE.

If there is a causal association, risk appears to increase with 25(OH)D levels below around 50 nmol/L (see Figure 5). The US Institute of Medicine recommends maintaining 25(OH)D levels of 50 nmol/L or higher [62] to ensure good bone health. If that level or higher was maintained through careful sun exposure, this may also achieve the potential benefits for MS through both vitamin D-dependent and vitamin D-independent pathways. Perhaps one question is how much evidence is needed before we act, for example to advise the public to reduce or stop smoking, or have regular short periods of sun exposure appropriate for skin type [63,64], particularly when that action is unlikely to cause harm [65]. Yet such messages would need to framed to ensure safety from the risks of skin cancer due to excessive sun exposure, and it may be difficult to change ingrained messages that have been effective in reducing skin cancer risks [66,67] and are well-known to the public.
